# Regorafenib: Antitumor Activity upon Mono and Combination Therapy in Preclinical Pediatric Malignancy Models

**DOI:** 10.1371/journal.pone.0142612

**Published:** 2015-11-23

**Authors:** Estelle Daudigeos-Dubus, Ludivine Le Dret, Claudia Lanvers-Kaminsky, Olivia Bawa, Paule Opolon, Albane Vievard, Irène Villa, Mélanie Pagès, Jacques Bosq, Gilles Vassal, Dieter Zopf, Birgit Geoerger

**Affiliations:** 1 Université Paris-Sud 11, Vectorology and Anticancer Therapeutics, UMR 8203, Villejuif, France; 2 CNRS, Vectorology and Anticancer Therapeutics, UMR 8203, Orsay, France; 3 Gustave Roussy, Vectorology and Anticancer Therapeutics, UMR 8203, Villejuif, France; 4 University Children’s Hospital, Department of Pediatric Hematology and Oncology, Münster, Germany; 5 PFEP (Plateforme d’évaluation préclinique), Gustave Roussy, Villejuif, France; 6 Tribvn, Châtillon, France; 7 Pathology Laboratory, Gustave Roussy, Villejuif, France; 8 Department of Neuropathology, Sainte-Anne’s Hospital, Paris, France; 9 Paris Descartes University, Paris, France; 10 Bayer Pharma Aktiengesellschaft, Berlin, Germany; University of Pécs Medical School, HUNGARY

## Abstract

The multikinase inhibitor regorafenib (BAY 73–4506) exerts both anti-angiogenic and anti-tumorigenic activity in adult solid malignancies mainly advanced colorectal cancer and gastrointestinal stromal tumors. We intended to explore preclinically the potential of regorafenib against solid pediatric malignancies alone and in combination with anticancer agents to guide the pediatric development plan. *In vitro* effects on cell proliferation were screened against 33 solid tumor cell lines of the Innovative Therapies for Children with Cancer (ITCC) panel covering five pediatric solid malignancies. Regorafenib inhibited cell proliferation with a mean half maximal growth inhibition of 12.5 μmol/L (range 0.7 μmol/L to 28 μmol/L). *In vivo*, regorafenib was evaluated alone at 10 or 30 mg/kg/d or in combination with radiation, irinotecan or the mitogen-activated protein kinase kinase (MEK) inhibitor refametinib against various tumor types, including patient-derived brain tumor models with an amplified *platelet-derived growth factor receptor A* (*PDGFRA)* gene. Regorafenib alone significantly inhibited tumor growth in all xenografts derived from nervous system and connective tissue tumors. Enhanced effects were observed when regorafenib was combined with irradiation and irinotecan against *PDGFRA* amplified IGRG93 glioma and IGRM57 medulloblastoma respectively, resulting in 100% tumor regressions. Antitumor activity was associated with decreased tumor vascularization, inhibition of PDGFR signaling, and induction of apoptotic cell death. Our work demonstrates that regorafenib exhibits significant antitumor activity in a wide spectrum of preclinical pediatric models through inhibition of angiogenesis and induction of apoptosis. Furthermore, radio- and chemosensitizing effects were observed with DNA damaging agents in *PDGFR* amplified tumors.

## Introduction

Angiogenesis plays a pivotal role in tumor growth and metastatic spread of solid malignancies [1]. High vascular endothelial growth factor (VEGF) expression is correlated with invasion, metastases and risk for recurrence in adult [2] and pediatric tumors [3–8]. Although antiangiogenic therapies inhibiting VEGF or its receptors VEGFR-1-3 appeared conceptually very promising, targeting VEGF alone has only shown limited efficacy in patients with solid tumors. Cancer neo-angiogenesis is a multifactorial process and is driven additionally by potential oncogenes like platelet-derived growth factor receptor (PDGFR) and/or fibroblast growth factor receptor (FGFR) signaling as reported in pediatric brain tumors such as medulloblastoma [9] and gliomas [9,10] and rhabdomyosarcoma [11,12], respectively. Furthermore PDGFRA represents an oncogene that is involved in cancer cell survival including pediatric glial tumours [13–16] as well as resistance to chemotherapy and radiation therapy, and its inhibition may sensitize tumors to DNA damaging agents [17].

Regorafenib (BAY 73–4506) is a new generation multi-tyrosine kinase inhibitor which potently inhibits angiogenic and oncogenic kinases, mainly VEGFR1-3, PDGFRA/B, FGFR1, Tie2, KIT, RET and BRAF[18]. Regorafenib showed potent inhibition of proliferation and migration in different adult cancers *in vitro* [19–21]. Induced anti-angiogenic effects were associated with significant antitumor activity *in vivo* as single agent in metastatic colorectal cancer, breast cancer, and glioblastoma models [18,22,23] and in combination with standard or new targeted therapies in hepatocellular carcinoma and metastatic colon cancer models [24,25]. Clinical phase I to III trials have shown activity in various advanced solid tumors and regorafenib was approved for metastatic colorectal cancer and gastro-intestinal stromal tumors (GIST) [26–30] following the CORRECT and the GRID pivotal trials respectively [31,32].

This study evaluates the therapeutic potential of regorafenib in preclinical models of solid pediatric tumors *in vitro* and *in vivo*. Its effect in combination with DNA damaging agents or other targeted drugs was investigated against xenografts derived from various tumor types.

## Material and Methods

### Drugs

Regorafenib (BAY 73–4506; Bayer Pharma AG) was stored as solid substance. For *in vitro* experiments, regorafenib was dissolved in 100% dimethyl sulfoxide (DMSO) and diluted in complete medium. For *in vivo* experiments, regorafenib was dissolved in propylene glycol, polyethylene glycol 400, and Kolliphor P188 (all Sigma-Aldrich) to a final concentration of 2 and 5 mg/mL. Refametinib (BAY 86–9766; Bayer Pharma AG) powder was dissolved in 30% 2-hydroxylpropyl-B-cyclodextrin Form 1. Solutions were freshly prepared every week. Irinotecan was purchased and diluted in saline.

### Cell lines

Regorafenib was screened on 33 solid tumor cells of the core cell line panel of the Innovative Therapies for Children with Cancer (ITCC) encompassing five medulloblastoma (DAOY, D283 MED, D341 MED, MED-MEB-8A, UW228.2), and seven each Ewing sarcoma (A673, EW7, ORS, POE, RD-ES, SIM(EW24), STA-ET-1), neuroblastoma (IMR-32, NGP, SH-SY5Y, SJ-NB-6, SJ-NB-8, SK-N-AS, SK-N-BE(2)), osteosarcoma (HOS, IOR-OS-9, IOR-OS-14, IOR-OS-18, MG-63, SAOS-2, U-2OS), and rhabdomyosarcoma (A204, RD, RH-18, RH-30, RH-41, RMS-1, RMS-YM). To note, the A204 cell line was recently reclassified as rhabdoid tumor cell line [33]. The adult TT thyroid carcinoma cell line, which comprises an activating mutation in the *RET*-proto-oncogene, was included as reference [18]. A673, DAOY, D283 MED, D341 MED, SK-N-AS, SK-N-BE(2), HOS, MG-63, SAOS-2, U-2OS, RD, TT were purchased from ATCC, RD-ES, IMR-32, NGP, SH-SY5Y, A204, RH-18, RH-30, and RH-41 from DSMZ. MED-MEB-8A was kindly provided by T. Pietsch (University of Bonn, Germany) [34], EW7, ORS, POE, SIM(EW24) by O. Delattre (Institut Curie, Paris, France) [35–37], SJ-NB-6, SJ-NB-8 by H. Caron (Amsterdam Medical Centre, The Netherlands) [38], IOR-OS-9, IOR-OS14, IOR-OS18 by M. Serra (Rizzoli Orthopaedic Institute, Bologna, Italy) [39,39], RMS-1 and RMS-YM by J. Shipley (The Institute of Cancer Research, Sutton, UK), all members of the ITCC consortium; UW228.2 was kindly provided by Dr. Silber (University of Washington, Seattle, USA) [40]. Luciferase gene transfected IGR-N91 cells were derived from metastatic neuroblastoma as reported previously [41]. Cell lines were maintained in Roswell Park Memorial Institute 1640 medium containing 10% fetal calf serum (FCS), penicillin (100 U/mL), streptomycin (100 μg/mL), and amphothericin B (0.25 μg/mL) at 37°C and 5% CO_2_, except IGR-N91-Luc which was maintained in Dulbecco‘s minimum essential medium Glutamax containing 10% FCS (all Life Technologies). Cell lines were regularly tested and found to be free of mycoplasma.

### MTS cell proliferation assay

Cells were seeded at 5,000 or 25,000 per well in 96-well plates. After 24 hours regorafenib was added to final concentrations between 0.01–100 μmol/L for 72 hours. Cell viability was determined using the CellTiter 96^®^ AQueous Non-Radioactive Cell Proliferation Assay (MTS assay; Promega) according to manufacturer’s instructions. Each concentration and time point was tested in quadruplet and all experiments in triplicates. Means and standard deviations were calculated from quadruple optical density (OD) measurements. Viability of treated cells was compared to that of untreated cells and drug concentrations which inhibited cell growth by 50% were calculated as described previously [42].

### Western blot analysis

Total tumor lysates were generated as previously described [43]. 30 μg of protein per sample were separated electrophoretically in 4–15% precast SDS polyacrylamide gels and transferred to precut polyvinylidene difluoride (PVDF) or nitrocellulose membranes using the Trans-Blot^®^ turbo^TM^ Transfer Starter System (all Bio-Rad). Chemiluminescence and colorimetric detection were performed using ChemiDoc^TM^ MP Imaging System and horseradish peroxidase conjugated mouse polyclonal anti-human PARP-1 (Ab-2, 1:600; Calbiochem), rabbit polyclonal anti-human p-ERK1/2 (Thr202/Tyr204), ERK1/2, p-AKT (Ser473), AKT, p-PDGFRA (Tyr762), PDGFRA, p-PDGFRB (Tyr751), PDGFRB, mouse monoclonal antibody anti-human β-Actin (S125; all 1:1000), detected with peroxidase-conjugated secondary anti-mouse or anti-rabbit antibody, respectively (1:5000; all Cell Signaling Technology) followed by chemiluminescence solution (Clarity™ Western Chemiluminescent HRP Substrate; Bio-Rad).

### Patient-derived human xenografts

Tumor samples and clinical information were collected with written informed consent (S1 Text.) of the parents/guardians before inclusion into protocols approved by the Internal Review Board of the Necker Sick Children's Hospital in Paris and the Gustave Roussy Cancer Institute in Villejuif.

Animal experiments were carried out under conditions established by the European Community (Directive 2010/63/UE) and approved by the CEEA26 Ethic Committee and the French Ministry (MENESR) (approval number: 00328.01). Xenografts were established from fresh tumor surgical excision. IGRM57, NEM14 and IGRG93 were established from a medulloblastoma in a 13-year-old boy, a high grade glioma in a 17-year-old girl, and a glioblastoma in a 69-year-old woman respectively, as described previously [44]. IGRM57 and IGRG93 were developed at Gustave Roussy in 1996 by Vassal et al. [45]. In the past, Informed consents were not required by French regulations; patients are now deceased, so consents are not available for these two models. NEM 14 was established at Gustave Roussy in 2006 from surgical tumor excision performed at Necker Enfants Malades hospital. Informed consent was signed by the parents and is available at Necker Enfants Malades.

### Experimental *in vivo* design

Experiments were approved by the CEEA26 Ethic committee and the French ministry (MENESR) (approval number: 00328.01) and carried out under conditions established by the European Community (Directive 2010/63/UE). Antitumor activity was evaluated against advanced stage tumors in female Swiss athymic mice of 6 to 8 weeks of age. Patient-derived xenografts were established by *in vivo* passages of 30 mm^3^ fragments [44], cell line xenografts by injection of 10^7^ cells into both flanks, orthotopic IGR-N91-Luc neuroblastoma by injection of 10^6^ cells in phosphate buffered saline into the left adrenal [41]. On day 0 before treatment started, animals bearing subcutaneous tumors of 80 to 300 mm^3^ were randomly assigned to treatment groups; treatment of the orthotopic IGR-N91-Luc model started three days after cell injection when positive bioluminescent signals were detected. Regorafenib was administered orally by gavage at 10 or 30 mg/kg daily for a minimum of 21 days; controls received vehicle. Irradiation was applied as total body irradiation of 1 Gy daily for five consecutive days using the gamma radiation source IBL 637 (CisBio, IBA). Irinotecan was administered intravenously at 27 mg/kg for 5 consecutive days, refametinib orally at 25 mg/kg/d for 5 days followed by 15 mg/kg/d. For combination treatments, regorafenib was administered 2 hours after irradiation, a minimum of 4 hours after refametinib, and concomitantly with irinotecan. Satellite mice which were treated equally to the therapy groups were sacrificed at earlier time points for pharmacodynamic analyses.

Clinical status of the mice was evaluated daily; body weight and tumor volumes were measured twice to three times per week. Tumor volumes of subcutaneous models were determined with a caliper and orthotopic models by weekly ultrasound [41,44]. The experiments lasted until tumor volumes had reached five times their initial tumor volume and a maximum of 2000 mm^3^ as endpoint. Mice were euthanized by CO2 gradient. Tumor doubling time (Td) was determined in an exponential growth phase between 200 and 400 mm^3^. Tumor growth inhibition (TGI) was calculated as the difference in mean tumor volume of treated tumors compared to controls at the day when the control group was terminated. Tumor growth delay (TGD) was defined as difference in median time to reach 5 times initial tumor volume compared to controls, complete tumor regression (CR) as tumor volume below palpation limit, partial regression (PR) as ≥50% decrease in volume in two consecutive measurements. Statistical significance was determined using the non-parametric Mann-Whitney or Kruskal-Wallis test and Prism^®^ software version 6.00.

### Histology and immunohistochemistry

Tumors were fixed in 4% paraformaldehyde and embedded in paraffin; 4μm sections were stained with hematoxylin-eosin-saffranin. CD34 immunohistochemistry and caspase 3 expression were determined after heat-induced antigen retrieval using rat anti-mouse CD34 antibody (1:20; Hycult technologies) and rabbit anti-rat antibody (1:400; Southern Biotech) and cleaved caspase 3 antibody (1:100; Cell Signaling), visualized by peroxidase/diaminobenzidine Rabbit PowerVision kit (ImmunoVision Technologies).

Representative whole tissue section from each animal was digitized using a slide scanner NanoZoomer 2.0-HT (C9600-13, Hamamatsu Photonics). Quantification of CD34-stained microvessels, apoptotic cells with cleaved caspase 3 and necrotic surface areas was performed using Calopix software (all Tribvn, 2.10.14, Morphometry 2.10.10 and vessels). Microvessel area profiles were expressed as vessel area fraction (%) of tumor tissue, apoptotic index as the number of brown staining nuclei per mm^2^, percentage of necrosis as ([total tumor area–total area without necrosis]/total tumor area) x 100.

### Fluorescence *in Situ* Hybridization

FISH was performed on interphase nuclei on zinc-formalin-fixed paraffin-embedded tissue using specific fluorescent probes for PDGFRA and centromere of chromosome 4 as reference (Abnova), counterstained with 4,6-diamidino-phenyl-indole (DAPI), according to optimized manufacturer’s instructions. Results were recorded using a DM6000 imaging fluorescence microscope (Leica Biosystems), CCD camera, and digital imaging software from Leica (CytoVision, v7.4).

## Results

### Regorafenib exhibits antiproliferative activity in pediatric tumor cell lines

**Fig 1:**
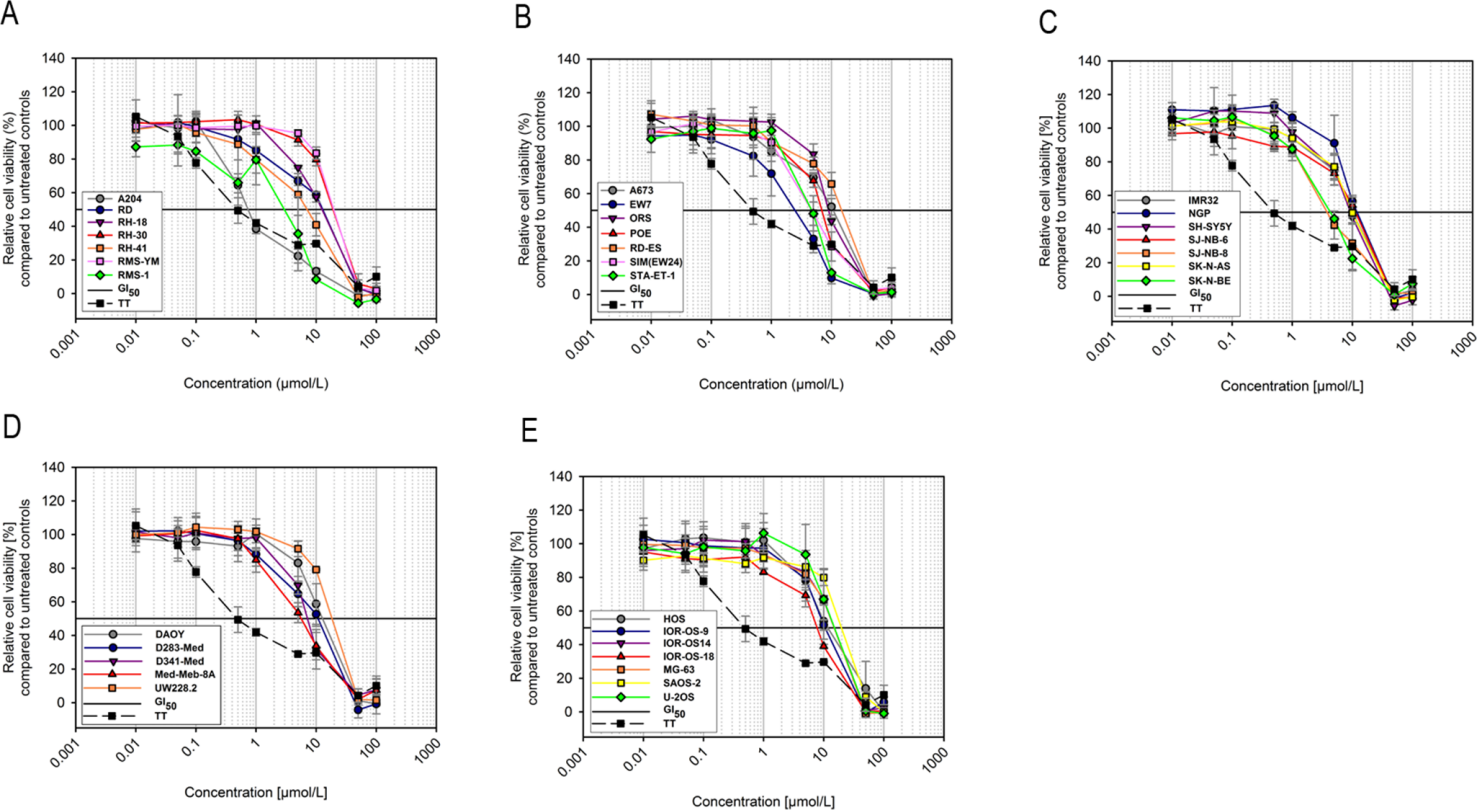
Inhibition of proliferation of pediatric solid tumor cell lines *in vitro* by regorafenib. 33 ITCC pediatric rhabdomyosarcoma (A), Ewing sarcoma (B), neuroblastoma (C), medulloblastoma (D) and osteosarcoma (E) cell lines were exposed to regorafenib at the indicated concentrations and cell viability was measured after 72 hours by MTS assay. The adult *RET* mutated TT thyroid cancer cell line is included as reference. Dose-response to regorafenib of all cell lines is plotted as the percentage of relative cell viability compared to untreated controls.

First we explored antiproliferative effects of regorafenib *in vitro* against 33 pediatric solid tumor cell lines (Fig 1; Table 1). Regorafenib inhibited cell growth in a dose-dependent manner. Concentrations, which reduced cell growth by 50% (GI_50_s), ranged from 0.7 μmol/L to 28 μmol/L with an overall mean of 12.5 μmol/L. A204 rhabdoid was the most sensitive with a mean GI_50_ of 0.8 μmol/L; 5 out of 7 Ewing sarcoma (EW7, ORS, POE, SIM, STA-ET-1), 2 out of 6 rhabdomyosarcoma (RH-41, RMS-1), 2 out of 5 medulloblastoma (D341 Med, Med-Meb-8A), 3 out of 7 neuroblastoma (SJ-NB-8, SK-N-BE(2), SH-SY5Y), and 1 out of 7 osteosarcoma cell lines (IOR-OS-18) presented with mean GI_50_s below 10 μmol/L.

**Table 1:** GI_50_s (μmol/L) of regorafenib in ITCC cell lines after 72h exposure determined by MTS Assay.

Cell Line	GI_50_ [μmol/L]	Mean ±SD per
	Mean ± SD	tumor type
**Ewing Sarcoma**		
A673	12. 1 ± 2.6	
EW7	3.2 ± 1.2	
RD-ES	19.5 ± 3.2	
SIM(EW24)	5.0 ± 1.1	8.7 ± 3.6
POE	7.3 ± 0.4	
ORS	9. 2 ± 0.3	
STA-ET-1	4.9 ± 0.7	
**Medulloblastoma**		
DAOY	15.2 ± 6.2	
D283 MED	14.2 ± 1.4	
D341MED	7.8 ± 0.8	13.7 ± 7.4
MED-MEB-8A	6.1 ± 1.3	
UW228.2	25.1 ± 0.3	
**Neuroblastoma**		
IMR32	10.7 ± 1.9	
NGP	14.4 ± 2.2	
SH-SY5Y	9.7 ± 1.3	
SJ-NB-6	12.8 ± 3.1	9.8 ± 3.8
SJ-NB-8	5.6 ± 2.8	
SK-N-AS	11.1± 2.0	
SK-N-BE	4.6 ± 0.1	
**Osteosarcoma**		
HOS	13.8 ± 5.4	
IOR/OS-9	11.5 ± 1.9	
IOR/OS-14	19.9 ± 1.1	
IOR/OS-18	8.2 ± 0.3	17.0 ± 6.8
SAOS-2	26.8 ± 1.8	
MG-63	20.0 ± 0.4	
U-2OS	18.8 ± 8.3	
**Rhabdomyosarcoma**		
A204	0.8 ± 0.1	
RD	16.2 ± 1.2	
RH-18	26.0 ± 0.5	
RH-30	15.5 ± 2.1	2.24 ± 2.64
RH-41	7.0 ± 2.4	
RMS-1	2.9 ± 0.2	
RMS-YM	26.7 ± 0.9	
**Reference cell line**		
TT	0.5 ± 0.2	

Overall, selected pediatric tumor cell line panel revealed a moderate sensitivity to regorafenib *in vitro* as compared to the TT thyroid carcinoma reference cell line which exhibited a mean GI_50_ of 0.5 μmol/L.

### Regorafenib exhibits significant antitumor activity against various pediatric cancer cell line derived xenografts

Based on the *in vitro* activity, cell lines were chosen from different tumor types to be evaluated in subcutaneous models (RMS-1 rhabdomyosarcoma, EW7 and STA-ET-1 Ewing sarcoma, SJ-NB-8 and SK-N-AS neuroblastoma).

**Fig 2:**
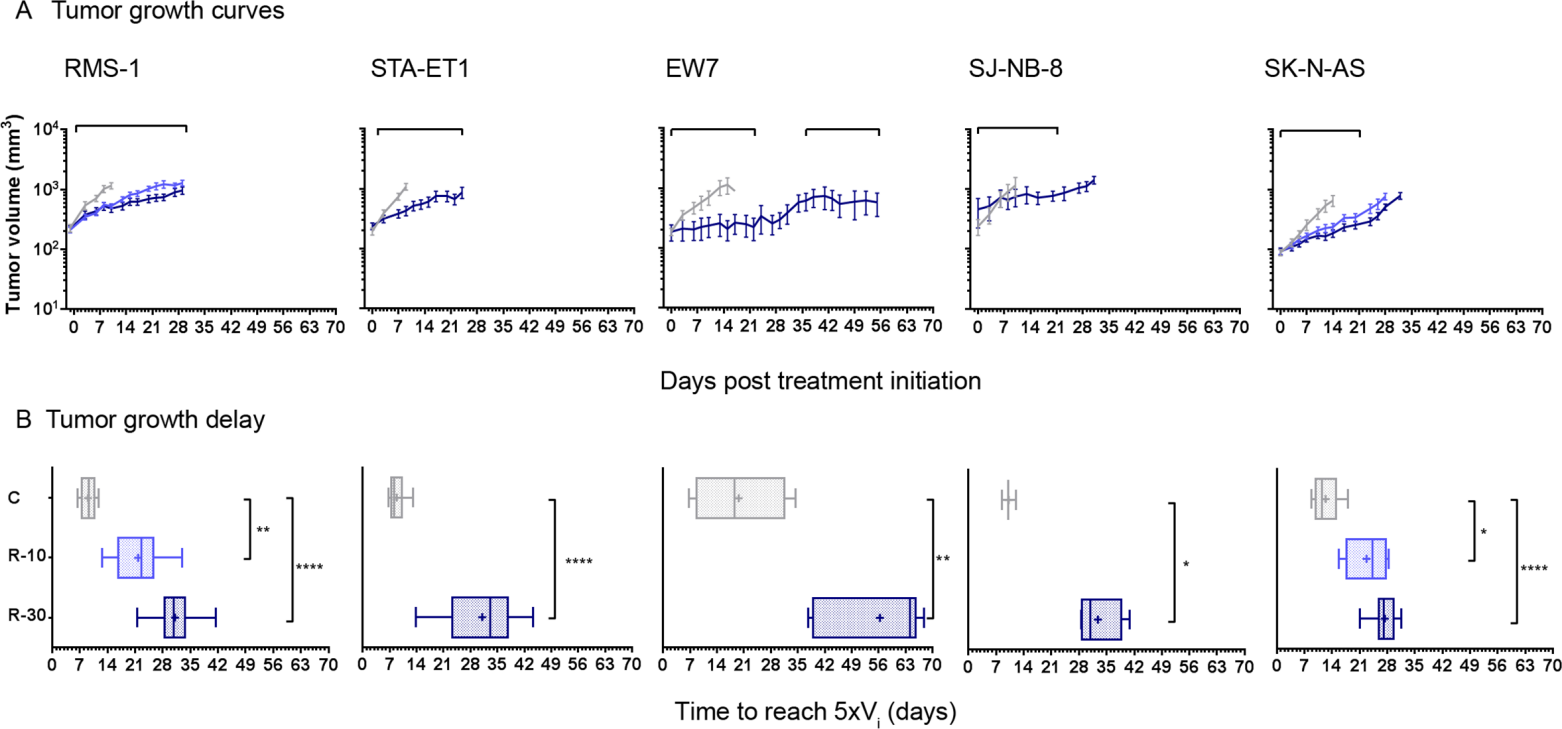
Antitumor activity of regorafenib *in vivo* against various subcutaneous pediatric tumor xenografts. Animals bearing subcutaneous RMS-1 rhabdomyosarcoma, STA-ET-1 and EW7 Ewing sarcoma, SJ-N-B8 and SK-N-AS neuroblastoma xenografts were treated orally with regorafenib at 10 mg/kg/d (light blue; R-10) and/or 30 mg/kg/d (dark blue, R-30), or with vehicle (grey, C) for a minimum of 21 days; treatment periods are indicated by bars above the graphs. (A) Graphs show arithmetic means ± standard error of mean (SEM) of tumor volumes. (B) Times to reach 5 times the initial volume (V_i_) of 3–14 tumors per group is displayed as box plot, + represents means and error bars minimum to maximum values. Statistical significance was estimated by Mann-Whitney or Kruskal-Wallis tests, ****p<0.0001 ***<0.001, **<0.01., *<0.05.

Regorafenib administered orally at dosages of 10 mg/kg/d and/or 30 mg/kg/d resulted in tumor growth inhibition (TGI) ranging from 73% to 93% and in significant tumor growth delay (TGD) determined by the median time to reach 5 times the initial tumor volume (>15 days for 30 mg/kg groups) in all subcutaneous xenografts tested (Fig 2; Table 2). The EW7 model regrew after treatment had been stopped and it responded again upon reintroducing regorafenib treatment, suggesting that it did not acquire secondary resistance. We also evaluated regorafenib in combination with the MEK inhibitor refametinib since MEK activation by MAPK14 was proposed to be involved in tumor resistance to sorafenib [46]. RMS-1 was chosen for this experiment because it expresses considerable levels of activated ERK1/2. Regorafenib alone resulted in a dose-dependent TGI at 10 and 30 mg/kg/d of 67% and 78% (p = 0.0058 and <0.0001, respectively, Kruskal-Wallis test) and a TGD of 13 and 22 days (p = 0.0036 and p<0.0001, respectively). Refametinib given alone at 25 mg/kg/d for the first 5 days followed by a dose reduction to 15 mg/kg/d (due to body weight loss) exhibited no significant TGI (19%) despite notable inhibition of ERK1/2 phosphorylation in tumors (S1 Fig). Its combination with regorafenib at 10 mg/kg/d enhanced growth inhibiting effects to 73% TGI (p = 0.0005) and the TGD to 23 days (p<0.0001) which was similar to the highest dose of regorafenib when given alone (Table 2).

**Table 2:** Anti-tumor activity of regorafenib alone and in combination against pediatric xenografts

Treatment	Schedule (dose dq)	Tumor (n)	Initial Tumor Volume (mm^3^)	Td (days)	PR	CR	TFS	TGI (at Day)	p	5 x Vi (days)	TGD (days)	p
**ET-1**												
** **								*D13*				
Control	0.15 mL x 15d	13	166 (75–426)	3.2	0	0	0			8		
Regorafenib	30 mg/kg x 30d	14	221 (93–473)		0	0	0	78%	0.0002	33	25	<0.0001
**EW7**												
** **								*D17*				
Control	0.15 mL x 24d	6	192 (80–250)	4.9						18.5		
Regorafenib	30 mg/kg x 21d x 2 cycles	6	203 (80–414)		0	0	0	93%	0.0152	64	46	0.0022
**SJ-N-N8**												
** **								*D16*				
Control	0.15 mL x 16d	3	180 (156–377)	3.7	0	0	0			10		
Regorafenib	30 mg/kg x 21d	4	242 (198–931)		0	0	0	82%	0.0286	31	21	0.0286
**SK-N-AS**												
** **								*D20*				
Control	0.15 mL x 20d	9	93 (37–143)	6.3	0	0	0			11.5		
Regorafenib	10 mg/kg x 21d	11	95 (45–136)		0	0	0	80%	0.008	25	13	0.0124
Regorafenib	30 mg/kg x 21d	10	85 (53–190)		0	0	0	87%	0.003	27	16	<0.0001
**RMS-1**												
** **								*D13*				
Control	0.15 mL x 13d	13	184 (75–470)	4.7						9		
Regorafenib	10 mg/kg x 34d	13	203 (113–332)		0	0	0	67%	0.0058	22	13	0.0036
Regorafenib	30 mg/kg x 44d	14	175 (74–420)		0	0	0	78%	<0.0001	31	22	<0.0001
Control combination	0.125 mL x 13d + 0.25ml x 5d, 0.15 mL x 8d	12	189 (137–397)		0	0	0	2%	ns	9		ns
Refametinib	25 mg/kg x 5d, 15 mg/kg x 8d	13	176 (73–480)		0	0	0	19%	ns	10	1	ns
Regorafenib + Refametinib	10 mg/kg x 38d + 25 mg/kg x 5d,15 mg/kg x 33d	13	162 (92–375)		0	0	0	73%	0.0005	32	23	<0.0001
**IGR-N91-Luc**												
** **								*D29*				
Control	0.15 mL x 29d	7		1.7								
Regorafenib	30 mg/kg x 29d	7						99%	0.0022			
**IGRG93**												
** **								*D11*				
Control	0.15 mL	10	156 (83–307)	2.9						9		
Regorafenib	10 mg/kg x 21d	10	140 (60–391)		0	0	0	86%	ns	27	19	0.0356
Regorafenib	30 mg/kg x 21d	11	200 (67–351)		0	0	0	101%	0.0408	32	23	<0.0001
Control + Radiotherapy	0.15 mL x 21d + 1 Gy x 5d	11	160 (62–311)		4	0	0	100%	ns	19	10	ns
Regorafenib + Radiotherapy	10 mg/kg x 21d + 1 Gy x 5d	10	215 (73–329)		6	3	1	111%	<0.0001	34	25	<0.0001
Regorafenib + Radiotherapy	30 mg/kg x 21d + 1 Gy x 5d	11	195 (68–500)		5	6	0	114%	<0.0001	37	28	<0.0001
**IGRM57**												
** **								*D27*				
Control	0.15 mL x 29d	6	285 (87–385)	3.7						19		
Regorafenib	30 mg/kg x 39d	6	217 (167–262)		5	0	0	108%	0.0043	62	43	0.0307
Control + CPT-11	0.15 mL x 39d + 27 mg/kg x 5d	7	231 (97–287)		5	2	0	108%	0.0012	55	36	ns
Regorafenib + CPT-11	30 mg/kg x 39d + 27 mg/kg x 5d	7	222 (125–444)		1	6	0	111%	0.0012	90	71	0.0005
**NEM14**												
** **								*D28*				
Control	0.15 mL x 29d	6	117 (79–166)	9	0	0	0			20		
Regorafenib	30 mg/kg x 29d	7	159 (80–184)		0	0	0	74%	ns	32	12	ns
Control + Radiotherapy	0.15 mL x 29d + 1 Gy x 5d	7	109 (80–171)		0	0	0	74%	ns	30	10	ns
Regorafenib + Radiotherapy	30 mg/kg x 29d + 1 Gy x 5d	7	150 (91–276)		0	0	0	93%	0.0176	42	22	0.0036

CR, complete regression; PR, partial regression; TGD, tumor growth delay; TGI, tumor growth inhibition; 5xVi, Time to reach five time initial tumor volume; TD, tumor doubling time; TFS, tumor free survivor at day 120; ns, non-significant; Statistical analysis was performed using the non-parametric Mann-Whitney test for experiments with 2 treatment groups and the Kruskal-Wallis test for those with more than 2 groups

Thus regorafenib significantly inhibits tumor growth in various pediatric malignancies independent of tumor histology types.

### Antitumor activity of regorafenib is mediated by anti-angiogenic effects and induction of apoptotic cell death potentially involving PDGFR

**Fig 3:**
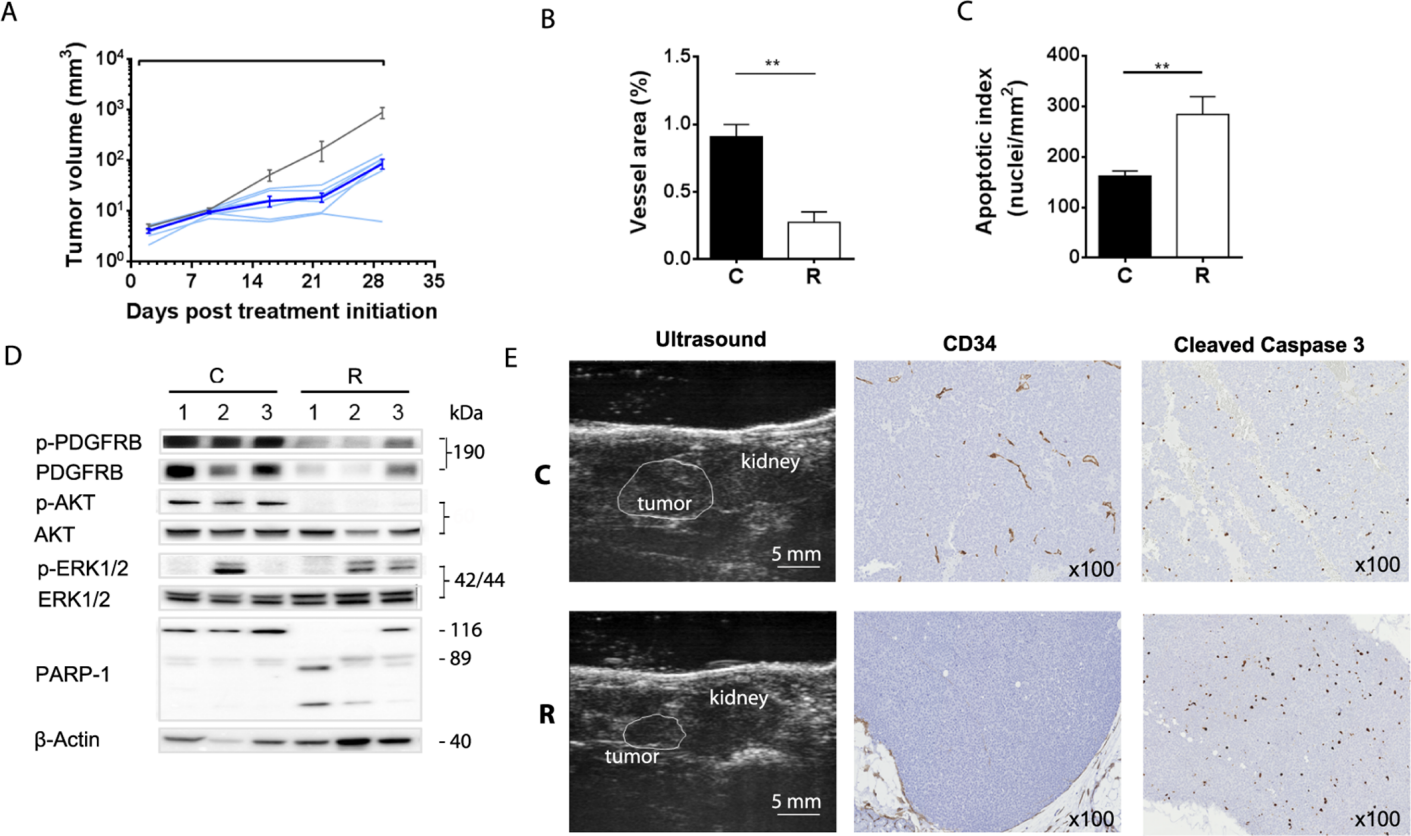
Regorafenib inhibits orthotopic IGR-N91-Luc neuroblastoma through antiangiogenic effects and induction of apoptosis. Animals bearing orthotopic adrenal IGR-N91-Luc tumors were treated orally with regorafenib at 30 mg/kg/d (blue lines) or vehicle (grey line) for 28 days. (A) Graph shows growth curves of individual regorafenib treated mice and arithmetic means (± SEM) of tumor volumes of 7 untreated and 6 treated tumors. Tumor volumes were determined by ultrasonography and representative images of adrenal tumors and kidneys at Day 22 post treatment initiation are displayed (E). Paraffin-embedded entire sections of adrenal IGR-N91-Luc xenografts from Day 29 post treatment initiation were stained immunohistochemically with antibodies against the endothelial cell marker protein CD34 and the apoptosis marker cleaved caspase 3. (B) Microvessel area, quantified as number of CD34-positive vessel using Calopix software, is presented as means ± SEM of 6 controls and 4 treated tumors (p = 0.0095; Mann-Whitney). (C) Apoptosis index is determined as number of caspase 3 cleavage positive cells in 6 controls and 6 treated tumors (p = 0.0043). Examples of histological stainings are shown at 100x magnification. Positive staining appears as brown color (E). (D) Total lysates of three individual tumors from Day 29 post treatment initiation were subjected to Western blot analyses using antibodies against phosphorylated (p-) and non-phosphorylated PDGFR, AKT, ERK1/2 and against cleaved PARP-1. β-actin was used as reference. C: control, R: regorafenib.

Subsequently regorafenib was tested against an orthotopic adrenal neuroblastoma model established from IGR-N91-Luc cells that exhibited a GI_50_ of 0.8 μmol/L *in vitro* (data not shown). Regorafenib resulted in 99% TGI as compared to the control group at day 29 when treatment ended (Fig 3A; Table 2).

In order to gain insight into the mechanisms by which regorafenib inhibits tumor growth we investigated tumor sections immunohistochemically and total lysates of tumors harvested at day 29 for the inhibition of some of its key targets and kinases involved in their signaling pathway by Western blot. Regorafenib treated xenografts showed a reduced microvessel area compared to control tumors as determined by CD34 staining (Fig 3B and 3E; p = 0.019, Mann-Whitney test) indicating that inhibition of angiogenesis is involved in regorafenib induced antitumor effects. Moreover, an increased apoptotic index was noted in regorafenib treated adrenal tumors (Fig 3C and 3E; p = 0.0043). In Western blot analyses regorafenib resulted in a pronounced reduction of both total and phosphorylated PDGFRB protein levels in three tumor lysates analyzed (Fig 3D). This was associated with inhibition of AKT activation indicated by strongly reduced p-AKT levels and enhanced PARP-1 cleavage which was consistent with an elevated number of cells positive for cleaved caspase 3 staining (Fig 3C–3E). PDGFRB receptors are highly expressed on IGR-N91 cells with normal copy numbers of the *PDGFRB* gene, and their regorafenib mediated reduction and its associated induction of apoptotic cell death suggest that PDGFRB signaling may be implicated in the survival of these cells and its inhibition may represent an additional mechanism by which regorafenib induced anti-tumor effects in this model.

### Regorafenib demonstrates broad antitumor activity in primary patient-derived xenografts which is enhanced with DNA damaging agents

**Fig 4:**
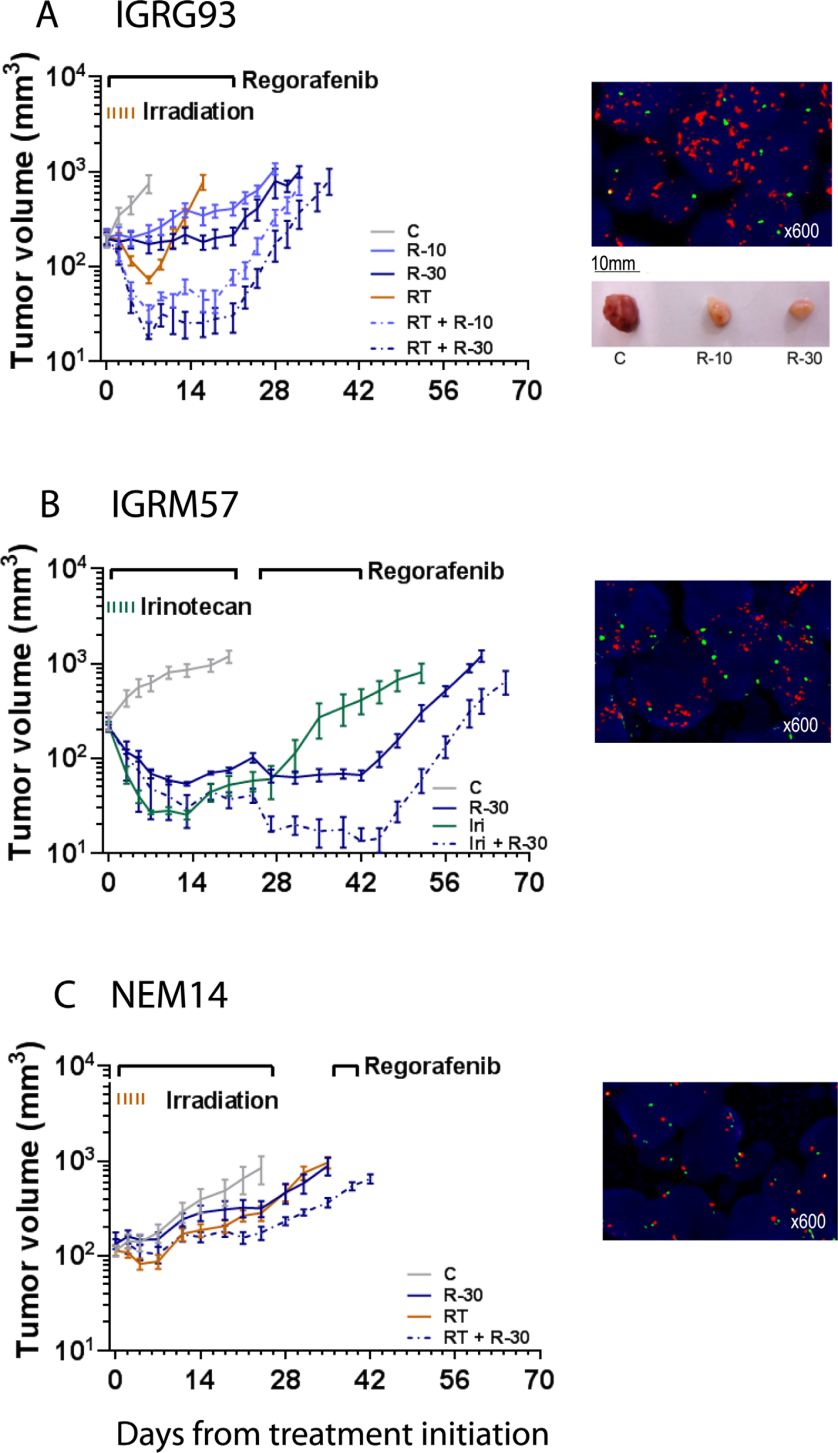
Antitumor activity of regorafenib alone and in combination against xenografts of *PDGFRA* amplified (IGRG93 and IGRM57), and non-amplified (NEM14) brain tumors. Animals bearing subcutaneous (A) IGRG93 and (C) NEM14 gliomas, and (B) IGRM57 medulloblastoma, were treated orally with regorafenib at 10 mg/kg/d (R-10) and/or 30 mg/kg/d (R-30) for a minimum of 21 days, total body irradiation of 1 Gy (RT) for 5 consecutive days (IGRG93, NEM14), or irinotecan at 27 mg/kg intravenously (Iri) for 5 consecutive days (IGRM57), respective combinations thereof as indicated, or vehicle (C). Graphs show arithmetic means ± SEM of tumor volumes of 6–14 tumors per group. Bars above graphs indicate treatment periods. Fluorescence *in situ* hybridization *for PDGFRA* gene amplification is shown in the right upper corner for each model (*PDGFRA* gene in red, centromere of Chromosome 4 in green); picture shows macroscopic aspect of IGRG93 tumors after 3 days of treatment.

PDGFRA is one of the main targets of regorafenib. We therefore set out to then examine if PDGFRA activation, due to amplification of the *PDGFRA* gene, an alteration frequently found in pediatric brain tumors (eg. gliomas and medulloblastomas) [13,14,47], affects regorafenib activity alone or in combination with DNA damaging agents. We performed these experiments in primary patient-derived brain tumor xenograft models with known molecular characteristics. The IGRG93 glioma carries a *PDGFRA* gene amplification (Fig 4A) and was included to sustain our hypothesis although the model derived from an adult glioma and no pediatric *PDGFRA* amplified model was available.

Treatment for 21 days with regorafenib at 10 and 30 mg/kg/d resulted in significant, dose-dependent TGI of 86% and 101%, respectively on Day 11, when the control group had to be terminated, and TGD of 19 and 23 days, respectively as compared to the control group (p = 0.0356 and p<0.0001; Kruskal-Wallis test; Table 2). When IGRG93 mice were irradiated with 1 Gy for 5 consecutive days, 4/11 partial regressions (PR) were detected; TGI was 100% and the TGD was 10 days but non-significant. Remarkably, when regorafenib was combined with irradiation an enhanced effect was observed resulting in 100% tumor regression. At a dose of 10 mg/kg/d, 4/10 complete regression (CR), including one animal which was tumor-free at 120 days, and 6/10 PR and at 30 mg/kg/d, 6/11 CR and 5/11 PR were observed. This TGI was accompanied by a significant TGD of 25 and 28 days respectively (both p<0.0001).

We speculated that enhanced effects of regorafenib may also occur with other DNA damaging agents and therefore evaluated its combination with the topoisomerase I inhibitor irinotecan, used in salvage strategies in brain tumors [48–50], against the pediatric IGRM57 medulloblastoma model, which also exhibits a *PDGFRA* amplification (Fig 4B). Regorafenib alone at 30 mg/kg/d resulted in 5/6 PR and irinotecan alone in 2/7 CR and 5/7 PR. The combination of both showed enhanced activity with 6/7 CR and 1/7 PR. Significant TGD of 43 days was observed for regorafenib alone which was further extended to 71 days in the combination treatment (p = 0.0307 and p = 0.0005 respectively, Kruskal-Wallis test; Table 2).

The enhanced effect of the combination of regorafenib with DNA damaging agents may depend on the *PDGFRA* amplification in these two brain tumor models. To test this, we evaluated regorafenib in combination with radiotherapy against the *PDGFRA* non-amplified pediatric glioma NEM14 (Fig 4C). Both single agent treatments, regorafenib at 30 mg/kg/d and irradiation inhibited tumor growth by 74% and delayed its progression by 12 and 10 days, respectively. For their combination TGI and TGD were enhanced to 94% and 22 days (p = 0.0176 and p = 0.0036, respectively, Kruskal-Wallis test; Table 2) however no tumor regression was observed. Thus potent anti-tumor activity with complete tumor regressions was observed in combination therapies with regorafenib and DNA damaging agents in *PDGFR* amplified models.

### PDGF receptors appear as potential mediators of enhanced cell death of the combination of regorafenib with DNA damaging agents

To uncover the mechanisms by which regorafenib inhibits tumor growth alone and in combination with DNA damaging agents we performed similar immunohistochemical and Western blot analyses as described above for the neuroblastoma model. Here, tumors that were harvested at day 3 of treatment were used because of the rapid tumor regression observed in these models.

**Fig 5:**
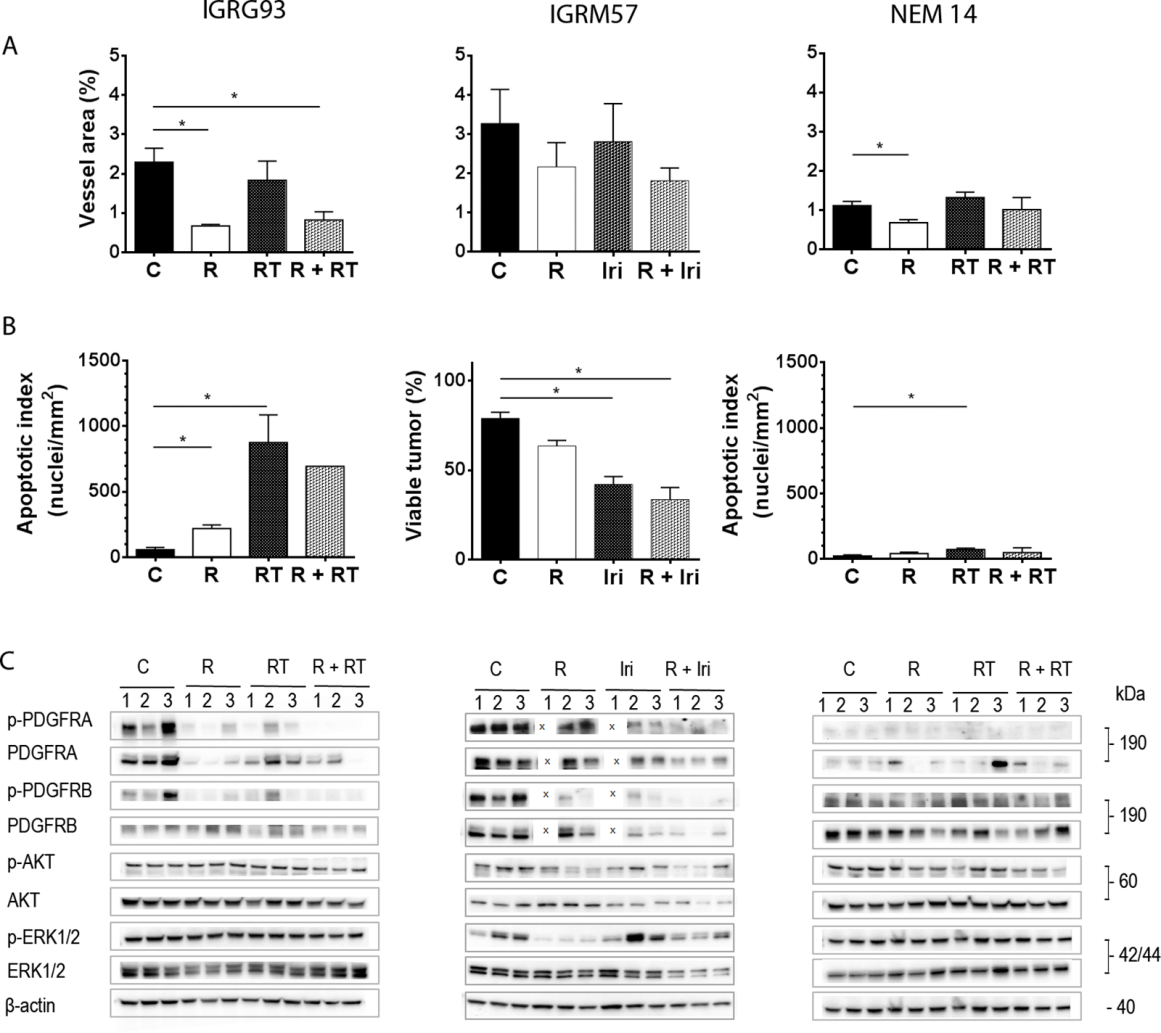
Regorafenib activity alone and in combination against patient-derived tumor models is mediated by anti-angiogenic effects and induction of cell death. Paraffin-embedded sections of IGRG93 (left column), IGRM57 (central column) and NEM14 (right column) tumors, harvested at Day 3 post-treatment initiation, were stained immunohistochemically with anti-CD34 and anti-cleaved caspase 3 antibodies and histologically with Hematoxylin-Eosin-Saffron (HES). (A) Microvessel area and (B) apoptosis index are presented as means ± SEM of 3 controls and regorafenib 30 mg/kg/d treated tumors each (*p<0.05; Mann-Whitney). Due to extensive necrosis only one sample could be evaluated in the combination group of the IGRG93 study. For IGRM57, the assessment of viable tumor by excluding necrotic areas was estimated on HES stained sections. (C) Total lysates from individual tumors were subjected to Western blotting for expression analyses of phosphorylated (p-) and non-phosphorylated PDGFRA, PDGFRB, AKT and ERK1/2. β-actin was used as reference. C: control, R: regorafenib; X: omitted sample due to limited availability of tumor 1; RT: irradiation, Iri: irinotecan.

In IGRG93 tumors (Fig 5, left column) regorafenib exerted a strong antiangiogenic effect indicated by a significant reduction of the microvessel area determined by CD34 staining (p = 0.05, Mann-Whitney test; Fig 5A). When apoptosis was evaluated, an increase of caspase 3 cleavage positive nuclei was noted in regorafenib-treated IGRG93 tumors as compared to the control group (p = 0.05) which, although significant, did not reach the increase observed in response to irradiation alone (p = 0.05; Fig 5B). It should be noted that the appearance of necrosis precluded the analysis in 2 out of 3 combined treated tumors. The amplification of the *PDGFRA* gene in IGRG93 leads to PDGF receptor A overexpression and its activation, when compared to NEM14 tumors, where the *PDGFRA* gene is not amplified. Regorafenib significantly reduced the levels of phosphorylated and total PDGFRA. In addition, regorafenib alone and in combination reduced phosphorylation of PDGFRB, which may also contribute to anti-tumor effects, however, without affecting AKT or ERK1/2 phosphorylation (Fig 5C).

The second *PDGFRA* amplified model IGRM57 (Fig 5, central column) was highly sensitive to regorafenib and irinotecan treatments. In IGRM57 tumors, which exhibited the highest degree of vascularization among all models analyzed, regorafenib slightly reduced the vessel area at this time point (Fig 5A). Caspase 3 cleavage induction was observed in regorafenib and irinotecan treated tumors, but could not be technically quantified in tumors of the combination group because of their extensive regression early on. Instead we compared the viable tumor surface area which was reduced most upon irinotecan treatment and when both drugs were combined as compared to controls (p = 0.05; Mann-Whitney test; Fig 5B). Untreated IGRM57 tumors overexpress activated PDGFRA receptor, which matches with the amplification of its gene in this tumor and which is similar to IGRG93. Overall PDGFRA levels were reduced upon combination treatment, but marginally affected by regorafenib alone, which differs from IGRG93. Well detectable levels of activated PDGFRB receptors were found in untreated tumors which were inhibited by regorafenib and even stronger by the combination. Inhibition of both receptors was associated with reduced levels of phosphorylated AKT and ERK1/2 (Fig 5C).

In NEM14 tumors (Fig 5, right column) regorafenib significantly reduced microvessel area but had no effect on the apoptotic index, which was only increased upon irradiation (both p = 0.05; Fig 5A and 5B). Expression levels of PDGFRA were only weakly detectable, consistent with the absence of gene amplification, and no notable effects were observed on both PDGF receptors, AKT and ERK1/2 kinases. These results suggest that the growth of NEM14 is mainly inhibited by the anti-angiogenic effect of regorafenib, which is consistent with the moderate tumor response.

Taken together tumor growth inhibiting activity of regorafenib is facilitated by its anti-angiogenic effects observed in all models. Importantly, deactivation of PDGFR signaling in models with an amplified *PDGFRA* gene appeared to be involved in the induction of tumor regressions when regorafenib is given in combination with DNA damaging agents.

## Discussion

Regorafenib was previously demonstrated to broadly inhibit tumor growth in preclinical models derived from adult tumors [18]. This is the first study which shows significant anti-tumor activity of regorafenib, alone or in combination with standard anticancer treatments, in a large panel of various pediatric solid malignancies.

When analyzed *in vitro*, regorafenib revealed dose-dependent anti-proliferative activity in a panel of 33 cell lines derived from five different pediatric solid tumor indications. Fifty per cent growth inhibiting concentrations ranging between 0.7 and 28 μmol/L were similar to those most often observed for regorafenib in tumor cell lines from adult carcinomas including multiple colorectal cancer cell lines [18,21], but higher than the concentration by which TT cells were inhibited. Regorafenib efficacy on the TT cells is explained by its potent inhibition of the activated RET kinase oncogene [18], which is the main driver of their proliferation. From our *in vitro* data we conclude that regorafenib does not target a kinase which is a driver for the proliferation of these pediatric solid tumor cell lines when cultivated as monolayers.

Subsequently we analyzed the antitumor effects of regorafenib *in vivo* in multiple pediatric tumor models originating from connective tissue (rhabdomyosarcoma, Ewing sarcoma) or tissues of the nervous system (neuroblastoma, glioma, medulloblastoma). They were derived from tumor cell lines or patient material and grown subcutaneously or orthotopically in mice. Regorafenib alone when dosed orally at 10 and 30 mg/kg/d, which results in an exposure in mice similar to the human exposure at the label dose of 160 mg per day [51], demonstrated significant antitumor effects in all models. Effects were dose-dependent, when tested. The extent of response varied among models whereby decelerated tumor progression compared to untreated controls was observed in most cases approaching disease stabilization in EW7 and IGRG93 at a dose of 30 mg/kg/d. Partial regressions were observed in IGRM57 tumors, which were previously seen with regorafenib only in preclinical GIST models carrying a mutated *KIT* oncogene (DZ, manuscript in preparation). Mutated *KIT* is a major driver of tumor growth in GIST cancer and is very potently inhibited by regorafenib [18]. The association between inhibitions of oncogenic kinases such as mutated RET and KIT and very potent antitumor activity suggests that regorafenib may also inhibit a tumor cell kinase which is critical for the growth of IGRM57 tumors. IGRM57 exhibits *PDGFRA* gene amplification though no hot spot mutations have been found in *PDGFR*, *RET*, or *KIT*. PDGFRs, which have been implicated in cancer [47,52,53] may play a role in this tumor, which is supported by the efficient inhibition not only of PDGFRB but also of its signaling kinases AKT and ERK1/2.

Based on the hypothesis that the *PDGFRA* oncogene, which is frequently present in pediatric brain tumors [13,14,47], may also be involved in sensitization to DNA damaging agents [17], we explored *in vivo* the combination of regorafenib with a topoisomerase I inhibitor and irradiation, both treatments commonly used for brain tumors in patients. For this purpose we selected three unique primary patient-derived xenograft models previously established and molecularly characterized at Gustave Roussy, which harbor amplifications of the *PDGFRA* gene, IGRG93 glioma derived from an adult glioblastoma and IGRM57 from a childhood medulloblastoma, or a normal copy number of the *PDGFRA* gene, NEM14 pediatric glioma. Intriguingly, enhanced benefit with hundred per cent tumor regression was observed in both *PDGFRA* amplified models which translates into elevated levels of activated PDGFRA receptors, either in combination with irinotecan in IGRM57 or irradiation in IGRG93. In contrast, no tumor regression, even not in the combination with irradiation, was found in the non-amplified *PDGFRA* NEM14, which in general responded less well to treatments compared to the other two models. These results suggest that regorafenib may act as a radiosensitizer similar to its related compound sorafenib [54,55] and as a chemosensitizer at least for topoisomerase I inhibitors, which was also previously observed in colorectal cancer models [21]. Additional genetic changes besides *PDGFRA* gene amplifications may be involved and further investigations are necessary.

Our investigation of regorafenib mechanisms of action was focused on the patient-derived models and was centered on angiogenesis inhibition, apoptosis induction and PDGFR signaling. We selected tumor xenograft models with distinct morphological and molecular characteristics in tumor vascularization and PDGFR expression due to *PDGFRA* gene amplification respectively independent of their histological type. Regorafenib alone exerted significant antiangiogenic effects in all patient-derived xenografts. The effect occurred in models with high (IGRM57) as well as low tumor vascularization (NEM14) with a rapid on-set by day 3 confirming previous observations by dynamic contrast-enhanced magnetic resonance imaging analyses [18,22] and maintenance for up to 29 days in the IGR-N91 model indicating its durability. Furthermore, in IGRM57 and EW7 we observed renewed tumor growth inhibition upon reintroducing regorafenib after treatment interruption suggesting that tumors had not acquired secondary resistance. DNA modifying agents had little if any effect on angiogenesis. Therefore the inhibition of tumor vascularization is considered as a major mechanism of action of regorafenib. Other tumor-cell derived xenografts including various sarcoma models were not studied in this respect, but we anticipate that this is also the case for these models. Indeed bevacizumab, an antibody against VEGF-A, was shown to delay tumor growth of a TC71 Ewing sarcoma xenograft in mice [56].

The effect of regorafenib alone on inducing apoptosis, although significant, was less pronounced resulting in a 2–3 fold increase versus control of the apoptotic index in IGRG93 and IGR-N91, which is similar to what was reported previously [22]. However its effect on the number of cells stained positive for cleaved caspase 3 in NEM14 was limited. In comparison irradiation increased the apoptotic index by 17-fold in IGRG93 and irinotecan reduced the viable tumor area to about 40% in IGRM57, indicating that these treatments exert their antitumor effects by apoptosis induction thereby significantly contributing to the combinatorial effect with regorafenib.

Analysis of main effectors of the complex PDGF signaling network [53] was performed to obtain initial insight into whether PDGFRs might play a role in these tumors. For this we have used total tumor lysates without considering differential expression of PDGFR in tumor epithelial cells or cells of the tumor microenvironment, such as pericytes, smooth muscle cells and cancer associated fibroblast [52]. A heterogeneous picture emerged from the Western blot analyses of the PDGFRs and their signaling kinases ERK1/2 and AKT, presumably reflecting the individuality of the four models and their tumor growth promoting mechanisms. In some models the PDGFR pathway may be involved for the following reasons. In untreated tumor lysates activated PDGFRB was expressed at well detectable levels in all patient-derived xenografts and activated PDGFRA in those where its gene is amplified. Their constitutive activation may cause and/or promote tumor growth as it was demonstrated by the induction of brain tumors through the PDGF-B like *v-sis* gene of the simian sarcoma virus in monkeys [57,58]. Recently, a case of a patient with colorectal cancer harboring strong PDGFR-β expression due to a newly described germline PDRFR-β mutation c.17C>T (NM_002609.3) was reported with substantial clinical response to regorafenib as compared to non-mutated colorectal cancer patients [59]. Regorafenib alone deactivated PDGFRB either by inhibition of its phosphorylation or even depletion of the entire protein, an effect which was enhanced in the combination, in all models except for NEM14. A similar effect was observed for PDGFRA in the IGRG93 tumors but was limited to the combination in IGRM57. The improved antitumor effects of the combination with radiotherapy and irinotecan in IGRG93 and IGRM57 could be explained by the inactivation of potentially oncogenic PDGFRs. Our data are consistent with the findings of the phase I clinical trial suggesting enhanced effects of irinotecan when combined with regorafenib in colorectal cancer [25].Intriguingly, PDGFR inactivation was associated with the inhibition of known receptor downstream effectors (i.e. AKT and ERK1/2 pathways) in IGRM57 and may promote the induction of apoptotic cell death as detected by increased PARP-1 cleavage (data not shown) and necrosis. In contrast no effects on AKT and ERK1/2 were found in IGRG93, despite significant tumor regressions, suggesting that there are different survival pathways involved. In NEM14 the overall treatment effects on the levels of phosphorylated or total kinases investigated were small suggesting that they do not play a role in the observed limited antitumor activity.

Although not studied here, PDGFR was implicated in the tumor development of Ewing sarcomas, which may add to the mechanisms by which regorafenib inhibits tumor growth in the EW7 model [60].

Although *in vitro* proliferation assays did not enable us to identify a specific pediatric indication with a particular susceptibility to regorafenib such as GIST or RET mutated thyroid cancer, we conclude from our *in vivo* results that regorafenib exhibits significant antitumor activity in a large variety of pediatric malignancies, independent of histological tumor type. This activity is primarily mediated through strong inhibition of angiogenesis. For the nervous system patient-derived xenografted models the inhibition of PDGFR signaling appears to be involved in single agent and particularly in combination strategies with DNA damaging agents which may be considered in the further development of this new compound.

## Supporting Information

S1 Fig Effect of regorafenib combined with refametinib against RMS-1 xenografts is associated with ERK1/2 inhibition. (A) Animals bearing RMS-1 rhabdomyosarcoma xenografts were treated orally with regorafenib at 10 mg/kg/d (R-10) or 30 mg/kg/d (R-30) for a minimum of 21 days, refametinib orally at 25 mg/kg/d for 5 days followed by 15 mg/kg/d (Ref) for 15 days, refametinib and regorafenib at 10 mg/kg/d combination (Ref+R-10), or vehicle (C). Graphs show arithmetic means ± SEM of tumor volumes of 11–14 tumors per group. The regorafenib single agent graphs are identical to those represented in Fig 2. (B) Total lysates from two to three individual tumors were subjected to Western blotting for expression analyses of phosphorylated (p-) and non-phosphorylated PDGFRA, PDGFRB, AKT and ERK1/2. β-actin was used as reference.
(EPS)

S1 Text Consent (PDF)
